# A 60 GHz Class-C Wide Tuning-Range Two-Core VCO Utilizing a Gain-Boosting Frequency Doubling Technique and an Adaptive Bias Scheme for Robust Startup

**DOI:** 10.3390/s25030981

**Published:** 2025-02-06

**Authors:** Ioannis Dimitrios Psycharis, Vasileios Tsourtis, Grigorios Kalivas

**Affiliations:** Department of Electrical and Computer Engineering, University of Patras, 265 04 Patras, Greece; vtsourtis@ece.upatras.gr

**Keywords:** mm-wave, class-C, VCO, CMOS, two-core, gain-boosted, frequency doubler, adaptive bias

## Abstract

This paper presents the design and the performance of a wide tuning-range millimeter-wave (mm-wave) two-core class-C 60 GHz VCO in 40 nm CMOS process, which can be integrated into wireless communication transceivers and radar sensors. The proposed architecture consists of a two-core 30 GHz fundamental VCO, a gain-boosted frequency doubler and an adaptive bias configuration. The two-core fundamental VCO structure achieves frequency generation in the vicinity of 30 GHz, where each VCO core targets a different frequency band. The two bands have sufficient overlap to accommodate for corner variations providing a large continuous tuning range. The desired frequency band is selected by activating or deactivating the appropriate VCO core, resulting in a robust switchless structure. This approach enables a considerably broad tuning range without compromising phase noise performance. Furthermore, the proposed topology utilizes an adaptive bias mechanism for robust start-up. Initially, the selected VCO core begins oscillating in class-B mode, and subsequently it transitions into class-C operation to offer improved performance. From post-layout simulations, after frequency doubling, the low-band VCO covers frequencies from 50.25 to 60.40 GHz, while the high-band VCO core spans frequencies from 58.8 to 73 GHz, yielding an overall tuning range of 36.92%. Owing to the gain-boosting topology, output power exceeds −14.2 dBm across the whole bandwidth. Simulated phase noise remains better than −92.1 dBc/Hz at a 1 MHz offset for all bands. Additionally, the two VCO cores never operate simultaneously, aiding in power efficiency.

## 1. Introduction

In recent years, there has been an increasing interest in designing and developing highly integrated millimeter-wave (mm-wave) transceivers in the 60 GHz frequency range [1]. The 60 GHz frequency band provides an expanded unlicensed spectrum. Initially, it was explored for high-speed wireless indoor communication, offering multi-Gb/s data rates. Over time, interest in this frequency band has expanded beyond wireless communication, with a growing focus on sensing applications [2,3]. Due to the high atmospheric attenuation at 60 GHz, which constrains the sensing range, this frequency band is well fitted for short-range frequency-modulated continuous-wave (FMCW) radar systems. These systems show promising potential for biometric sensors, healthcare monitoring and imaging for autonomous vehicles [4]. A frequency synthesizer is a critical component of an FMCW radar sensor, designed to provide a wide tuning range, low-phase noise for optimal detection sensitivity, and high output power to ensure proper functionality [4,5]. A Voltage-controlled oscillator (VCO) is a key component in FMCW radar frequency synthesizers [6]. In an FMCW radar sensor, the VCO generates the chirp signal in the transmitter chain and is utilized for mixing in the receiver to generate beat frequencies [7]. With its cost-effectiveness and high level of integration, CMOS process is widely employed for the implementation of various mm-wave transceivers [8]. In CMOS-operated mm-wave transceivers, specifically those working at 60 GHz and beyond, there is a significant demand for large tuning span, sufficient output power and low phase noise oscillators. These favorable VCO characteristics enhance the overall system performance, for instance in mm-wave FMCW radar systems, where a VCO offering extended tuning span alongside low phase noise leads to high range resolution and the best target discrimination, respectively [9,10]. However, despite their improved capabilities including high bandwidth and low silicon footprint, the most advanced CMOS technology nodes face significant challenges such as low-supply voltages and short channel effects arising from the technology scaling. Moreover, achieving a high purity, a large tuning range and high output power signal sources remains a challenging task. This stems from the inherent challenges posed by the low gain of CMOS transistors operating in close proximity to their unity-gain frequency ($f_{T}$), along with the drop in the quality factor of passive components. Furthermore, compared to alternative processes like SiGe HBT, the CMOS process is inferior regarding phase noise performance and delivered output power [11,12]. This article aims to enhance the tuning span of 60 GHz CMOS VCOs, offering moderate output power and preserving the phase noise performance by applying novel design techniques.

Mm-wave direct LO generation possesses several difficulties [13,14,15,16]. The output power of LC CMOS VCOs reduces with increasing frequency due to the increased parasitic effects. In addition, the elevated parasitic resistance of the metal interconnections due to the skin effect, alongside the degraded quality factor of the passive components, lead to elevated phase noise and power-hungry designs to meet loop gain requirements for proper start-up. Moreover, their tuning range is frequently constrained due to the presence of substantial parasitic capacitances. Additionally, the oscillation frequency is highly susceptible to variations from the manufacturing process, while the impact of parasitic elements becomes especially pronounced at mm-wave frequencies.

To overcome the aforementioned shortcomings of fundamental VCOs operating in the mm-wave regime, frequency multiplication is often employed [6,17,18]. By means of frequency multiplication, the fundamental VCO oscillates at a lower frequency relaxing most of the strict synthesizer requirements and then transitions to higher harmonics via frequency multiplication. Primarily, power consumption is greatly reduced as the transistors operate far away from the cut-off frequency, producing more gain, and thus the VCO achieves the required start-up loop gain with less power consumption. Phase noise improvement is also anticipated notwithstanding the 20log10N (dB) decline attributed to frequency multiplication. The multiplication factor not only expands the tuning range but also significantly mitigates sensitivity to parasitic elements and variations [19].

In recent years a plethora of studies have been published exploring frequency generation at 60 GHz. In [20], two fundamental 60 GHz VCOs with 15 dBm output power are proposed. While both VCOs demonstrated high output power, they also exhibited considerable power consumption. The first VCO employed a MOS varactor, achieving moderate phase noise and a wide tuning range. The second VCO employed a variable inductor (VID), achieving phase noise levels as low as 99 dBc/Hz at a 1 MHz offset, with a restricted tuning range of just 3%. In [21] researchers introduced a low-power VCO that operated within a frequency range of 59.8 to 65.4 GHz by utilizing transformer feedback techniques. The VCO achieved a phase noise of −94.9 dBc/Hz at a 1 MHz offset from 61.3 GHz and an output power of −10 dBm at the same frequency. A study in [22] presents a fundamental quad-core VCO with a frequency range spanning from 55.96 to 62.83 GHz. The proposed design demonstrated output power levels of about −4 dBm throughout the whole bandwidth, while phase noise varied from −95 dBc/Hz to −91.3 dBc/Hz at 1 MHz. The whole structure dissipated 15.3 mW. A wideband 60 GHz VCO is reported in [23]. The proposed structure incorporated a fundamental 30 GHz VCO, while the second harmonic signal is extracted from the center tap of the tank differential inductor. The 60 GHz final VCO consumed 4.2 mW and showed a tuning range of 10 GHz, from 55 to 65 GHz while phase noise stayed below −93.5 at 1 MHz across the whole frequency range. To overcome the issues of limited tuning range and high phase noise in fundamental VCOs at 60 GHz, the authors in [17] propose a 15 GHz VCO that utilizes fourth harmonic extraction to reach the 60 GHz frequency range. The 60 GHz signal was obtained by extracting the fourth harmonic from the tail node of the fundamental VCO. The design achieved a phase noise of −99.8 dBc/Hz at a 1 MHz offset from 56.1 GHz and offered an extended frequency range from 56.1 GHz to 65.6 GHz, along with moderate power consumption. A dual-core push-push voltage-controlled oscillator (VCO) is discussed in [24]. The fundamental VCO operated at 30 GHz, with its second harmonic signal being extracted from the oscillator’s tail using a transformer, delivering an output power of approximately −22 dBm. The proposed 60 GHz dual-core design achieved a phase noise of −96.8 dBc/Hz at a 1 MHz offset from the carrier frequency of 61.4 GHz and offered a wide tuning range of 18%, although it consumed a total power of 90 mW. From the above works, it is concluded that fundamental 60 GHz VCOs suffer from a reduced tuning span and, in most cases, elevated phase noise levels. On the other hand, VCOs that exploit higher harmonic generation present an extended tuning range and more competitive phase noise levels.

This paper introduces a 60 GHz generator spanning from 50.25 to 73 GHz, incorporating a dual-core fundamental class-C 30 GHz VCO, an adaptive bias topology to facilitate the oscillation start-up and a gain-boosted frequency doubler. Both VCO cores are coarse-tuned by an analog MOS varactor. The low frequency band VCO core utilizes a 4-bit binary weighted capacitor bank for fine frequency tuning, while the high frequency band VCO core is fine-tuned by a 3-bit binary weighted capacitor bank. This approach minimizes the gain of the fundamental VCO (K_VCO_) preserving phase noise performance. The two cores are activated/deactivated depending on the selected frequency band. The fundamental signal from each core is propagated to a push-push gain-boosted frequency doubler. The second harmonic signal is collected from the common drain node of the frequency doubler and subsequently is passed to the output pads through a balun which resonates near 60 GHz. Due to the effectiveness of the gain-boosting technique, output power always exceeds −14.2 dBm across the whole operational bandwidth of more than 20 GHz, whereas the unwanted harmonics are well below the desired second harmonic signal. Phase noise levels at the output vary from −99.2 to −92.1 dBc/Hz at 1 MHz offset from the carrier. Power consumption of the whole structure reaches 7.3 mW when the low-band VCO core is active and increases to 11.3 mW when the high-band VCO core is enabled. This paper is an extended version of a publication in the International Conference on Modern Circuits and Systems Technologies (MOCAST) 2024 [25] by the same authors. In this extended paper, both VCO cores have been slightly altered, and an adaptive bias circuitry has been designed to counteract the start-up problem in class-C VCOs, while the oscillation frequency has been doubled via a gain-boosted frequency doubler. The main contribution of this paper is to implement a large tuning range robust VCO operating in the 60 GHz band without compromising the phase noise performance and preserving power consumption at low levels.

The paper follows this structure: In Section 2, the design considerations regarding the fundamental two-core 30 GHz VCO are introduced, highlighting the operational principle of the proposed dual-core switchless architecture. Additionally, the impact of tank components on the phase noise performance is examined. Furthermore, the adaptive bias scheme which transforms both VCO cores from class-B to class-C is analyzed. Finally, the gain-boosting frequency doubling technique is investigated. Section 3 presents and compares simulation results with other state-of-the-art circuit proposals in the 60 GHz band. Finally, Section 4 concludes the paper.

## 2. Proposed Design

### 2.1. Proposed Architecture

**Figure 1:**
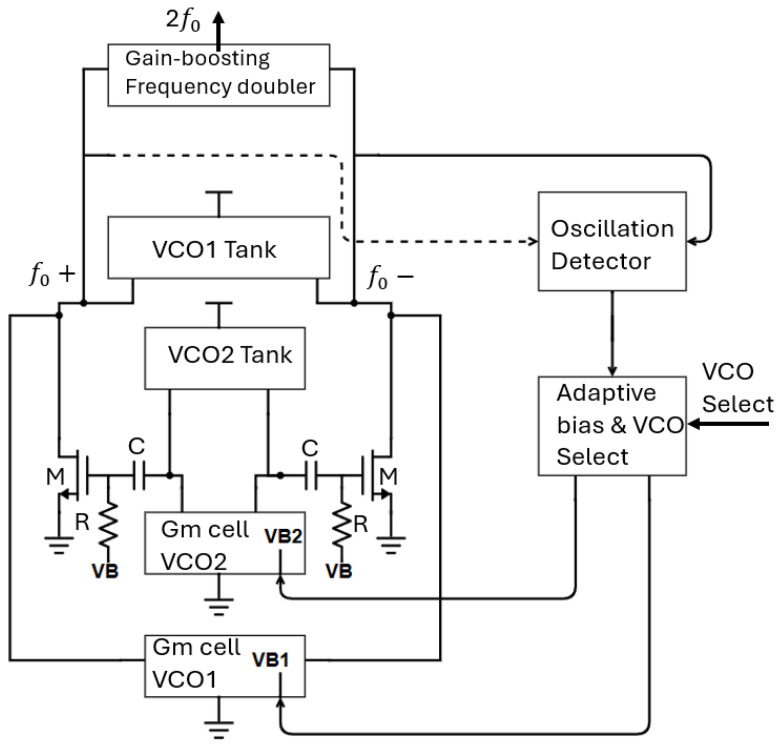
Proposed architecture.

The block diagram of the proposed topology is demonstrated in Figure 1. The whole structure includes two class-C VCO cores that are activated/deactivated by setting their bias voltages (VB_1_, VB_2_) to the appropriate value. VCO_1_ oscillates at a lower frequency band than VCO_2_. The selected VCO is selected by a VCO select control bit as shown in Figure 1. The high frequency lower band signal from VCO_1_ is directly fed to the gain-boosting doubler, while the RF signal from VCO_2_ is propagated to the frequency doubler through a common source amplifier (transistor M and bias network R, C) which uses the LC tank of VCO_1_ as a resonance load. The two cores never operate simultaneously leading to a power-efficient structure. The second harmonic signal near 60 GHz is passed to the output pads for measurement purposes via a balun. A control/select circuitry activates the desired oscillator core and deactivates the other. The selected VCO core begins oscillating in class B mode and subsequently maintains oscillations in class C mode. A detector circuit monitors the oscillator’s output amplitude and passes it to the adaptive bias circuitry, which generates a gate voltage to properly bias the selected oscillator core.

### 2.2. Fundamental VCO Considerations

#### 2.2.1. Topology Selection for the Fundamental LO Generation

**Figure 2:**
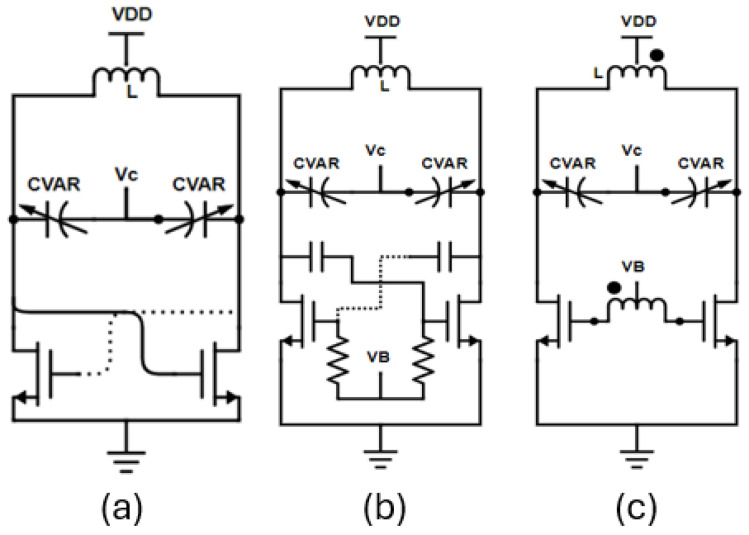
This is a figure presenting a typical class-B VCO and two commonly used class-C VCO topologies. (**a**) Typical class-B VCO, (**b**) the conventional class-C topology wherein (**c**) the gate biasing is provided through the center tap of the secondary winding of the transformer.

In mm-wave applications, LC Voltage-Controlled Oscillators (VCOs) are typically favored due to their robust and reliable performance. Figure 2 demonstrates two possible structures for an LC Class-B VCO. Both circuits consist of an NMOS cross-coupled pair, a varactor for the continuous frequency tuning and a differential inductor with a central tap. Figure 2a presents a classic class-B VCO where the gate and the drain of the NMOS transistors are biased to the same DC voltage throughout the central tap of the inductor. Even though this topology is mainly reliable and does not present start-up difficulties, in the case of a large voltage swing, the voltage at the gate of the transistor exceeds the voltage of the drain plus one threshold voltage indicating that the transistor is driven into the triode region [26]. Thus, the transistor produces less gain, and the phase noise performance deteriorates as the series resistance between the drain and the source of the transistor (R_ON_) appears in parallel with the tank resistance. Figure 2b illustrates the basic structure of a class-C VCO. The gate and the drain of the NMOS cross-coupled pair are biased separately. The appropriate gate biasing is provided throughout DC block capacitors and large resistors, and thus the transistor stays in saturation for a longer fraction of time. Finally, in Figure 2c the biasing of the gates of the NMOS pair is provided by the secondary middle tap of a dual-tapped transformer, avoiding the use of large capacitors in series with signal path which deteriorate the quality factor to some extent and are also prone to manufacturing variations. In the class-C operation, the active devices produce sharper current pulses to the tank due to the small conduction angle of the transistors [27]. This results in improved DC to RF efficiency. The class-C VCO exhibits a phase noise approximately 2 dB to 4 dB lower than a typical class-B VCO with the same power dissipation [28,29]. The major drawback of class-C VCOs is that the gate bias voltage directly affects the transconductance and hence the loop gain. A lower gate voltage due to manufacturing variations leads to start-up failure. For this reason, an adaptive bias circuitry has been designed to alleviate the start-up problem as will be shown below.

#### 2.2.2. Fundamental Two-Core VCO Design

**Figure 3:**
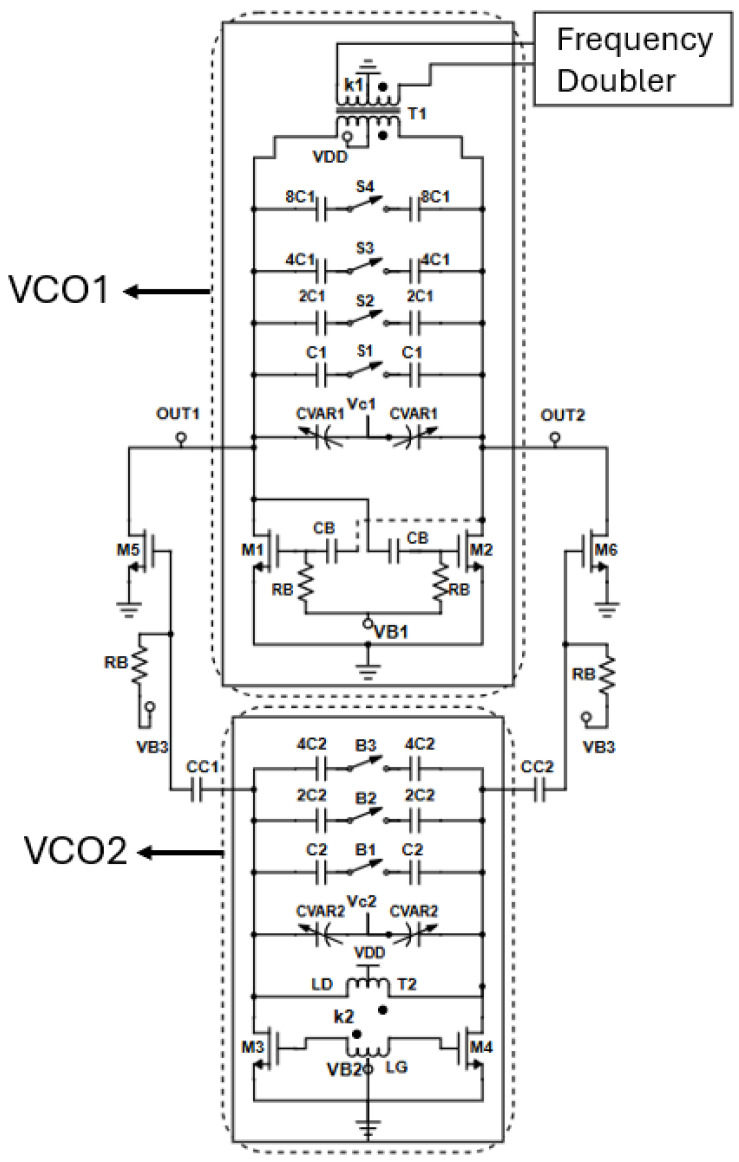
Proposed two-core fundamental VCO.

The electrical schematic of the proposed large tuning-range fundamental dual-core 30 GHz VCO is illustrated in Figure 3.

**Figure 4:**
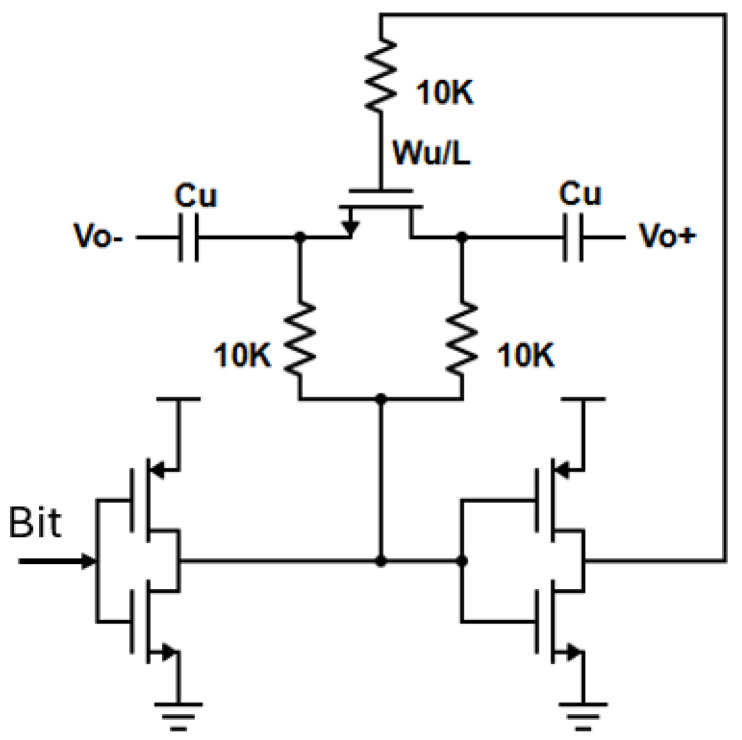
Unit switch structure implemented in both VCO cores.

The lower-band VCO (VCO_1_) oscillates between 25.13 and 30.2 GHz, whereas the higher-band VCO (VCO_2_) covers the frequencies between 29.4 and 36.5 GHz, leading to a total significant tuning range. The two VCO cores overlap by nearly 1.1 GHz to avoid dead zones and to guarantee frequency continuity. The lower-band VCO adopts the class-C capacitively coupled configuration, while the higher-band VCO adopts the class-C inductively coupled topology. The main reason for the capacitively coupled class-C configuration for VCO_1_ is the transformer (T1) which propagates the fundamental signal from both VCO cores to the frequency doubler. An inductively coupled class-C topology for VCO_1_ would lead to a triple coil transformer (T1) and, hence, a decreased quality factor for the whole structure. The LC tank in VCO_1_ consists of a four-bit binary weighted capacitor bank (C_1_–8C_1_) with a differential switch (S_1_–S_4_), leading to sixteen oscillation bands, alongside a differential dual-tapped transformer (T1), whereas VCO_2_ utilizes a 3-bit binary weighted capacitor bank (C_2_–4C_2_) with a differential switch (B_1_–B_3_), leading to eight oscillation bands, alongside a dual-tapped transformer (T2). The choice of a multi-bit capacitor bank in both oscillator cores instead of a large varactor is beneficial, as by dividing the total frequency range into smaller sub-bands, the VCO gain (K_VCO_) is reduced, rendering the VCO less sensitive to variations in the control voltage and/or the supply and also improving the phase noise performance [30]. The switch structure utilized in both capacitor banks is illustrated in Figure 4. The width (W_u_) of the switch transistor is crucial for the phase noise and tuning range performance. Both VCOs utilize an analog varactor (C_VAR1_, C_VAR2_) for continuous frequency tuning.

In both VCO cores, the cross-coupled pairs consist of RF low-threshold-voltage (LVT) NMOS transistors (M_1_–M_4_) with a gate width of 18 μm and minimum channel length of 40 nm. The gate finger width of both cross-coupled pairs has been set to 1 μm. Opting for a multi-finger transistor configuration greatly minimizes the unwanted gate resistance. In the entire circuit a high degree of symmetry is employed to reduce flicker noise-up conversion arising from minimum channel length devices. VCO_1_ has its gate biased by utilizing two DC-block capacitors (C_B_) alongside large biasing resistors (R_B_). The biasing capacitor (C_B_) needs to be large, as it forms a capacitive divider with the intrinsic capacitance between the gate and the source of the transistor (C_GS_) lowering the loop gain and causing startup problems, however, this comes at the cost of a lower quality factor. The 30 GHz signal from the output of VCO_1_ is fed to a gain-boosting frequency doubler via the secondary winding of transformer T1. The gates of the cross-coupled pair in the high-band VCO are biased through the center tap of transformer T2. The coupling coefficients k_1_ and k_2_ for both transformers are crucial for the performance, as will be analyzed below. In both VCO cores, the primary winding of the transformers (T1, T2) is connected to the supply voltage. The selected bias voltage (V_B1_ or V_B2_) at the gates of the cross-coupled pair of the selected VCO core is initially set to the supply voltage to start the oscillation in class-B mode. Afterwards, the adaptive bias topology lowers the gate bias voltage and steadies the selected core to class-C mode. Each VCO core is activated/deactivated by the VCO select unit as depicted in Figure 1.

**Figure 5:**
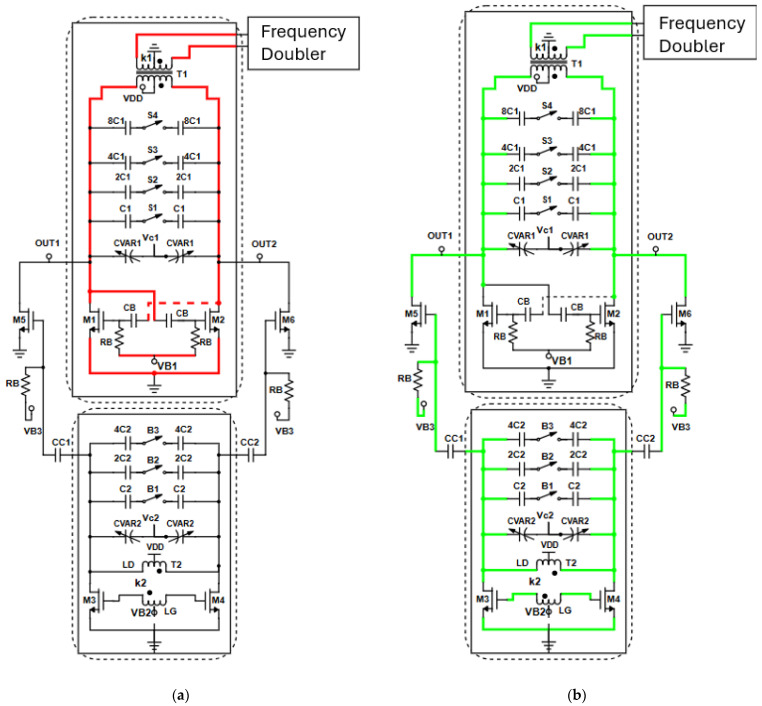
Activation/deactivation process for each VCO core. (**a**) The signal path when the low band VCO is turned ON; (**b**) the signal flow while the high band VCO is turned ON.

The activation/deactivation operation for both cores is illustrated in Figure 5. When the lower band VCO is selected to operate, the bias voltage V_B1_ is set up to the supply voltage by the VCO select unit and the bias voltage of the high band VCO is set to zero. This is illustrated in Figure 5a, where the high frequency signal path is highlighted by the red line. The fundamental signal is directly propagated to the frequency doubler via the secondary winding of transformer T1. For the activation of the high frequency band VCO, V_B1_ is set to zero and V_B2_ is set to the supply voltage. In this case, the signal follows the green line in Figure 5b. The 30 GHz signal from VCO_2_ passes through the frequency doubler through an AC-coupled common-source buffer amplifier (M_5_, M_6_), which uses as a resonant load the primary winding of transformer T1, the capacitor bank and the varactor of the low-band VCO, leading to a compact and area saving design. Instead of biasing the transistors (M_5_, M_6_) to the supply voltage through the drains of the high-band VCO core which results in expansive power dissipations, they are biased through an RC-network of C_CC1_, C_CC2_ and R_B_ preventing energy conservation. The high frequency signal is finally passed to the frequency doubler through the secondary winding of transformer T1. To maximize the conversion gain of the common source buffer (transistors M_5_, M_6_), the capacitance of the tank of the low-band VCO1 is set at its minimum value. Each activated VCO core dissipates 6.8 mW while the common source buffer consumes an extra 4 mW, although it is activated by the bias voltage V_B3_ only when VCO_2_ is turned ON.

Table 1 summarizes the parameters of both the low band and the high band VCO cores. The design employs rotative metal-oxide-metal capacitors.

#### 2.2.3. Phase Noise Considerations

The main contributors to the phase noise performance are given below by Leeson’s formula below:

(1) $$L\left(\Delta \omega \right)=\frac{4Fk_{B}TR_{Tank}}{A^{2}}{\left(\frac{\omega_{0}}{2Q_{Tank}\Delta \omega }\right)}^{2}$$

where $\omega_{0}$ is the output frequency of the signal source, Q_Tank_ is the tank quality factor, R_Tank_ is the parasitic parallel tank resistance, Δω is the frequency offset from the carrier frequency, F is the noise factor, k_B_ is Boltzmann’s constant, T is the absolute temperature and A is the peak output voltage. From Equation (1), to improve the phase noise, only the quality factor of the tank and signal output voltage can be altered through the design. In addition to that, the derivative of the frequency to the control voltage (K_VCO_) also contributes to phase noise. A larger K_VCO_ leads to a larger AM-PM conversion deteriorating the phase noise. To expand the tuning range of each VCO core and keep K_VCO_ as small as possible a multibit binary weighted capacitor bank is utilized for both cores.

**Figure 6:**
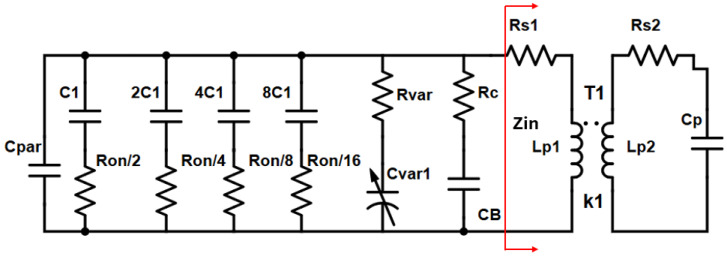
LC tank of the low-band VCO core (VCO_1_).

The total quality factor of each VCO core tank is given by Equation (2) below:

(2) $$\frac{1}{Q_{tank}}=\frac{1}{Q_{L}}+\frac{1}{Q_{C}}$$

where Q_L_ stands for the quality factor of the inductive components connected to the tank and Q_C_ for the quality factor of the capacitive elements connected to the tank. In Figure 6 the tank of the low band VCO (Figure 1, VCO_1_) core is illustrated.

In Figure 6, C_var1_ stands for the varactor capacitance, R_var_ is the series parasitic resistance of the varactor, C_par_ is the parasitic capacitance of the tank, including the parasitic capacitance of the cross-coupled pair (M_1_, M_2_), the parasitic capacitance of the buffer transistors (M_5_, M_6_), the parasitic capacitance of biasing resistors R_B_ to the substrate and the parasitic capacitance of the switches (S_1_, S_4_). R_C_ stands for the series parasitic resistance of the C_B_ capacitor used in VCO_1_. Furthermore, Ron/2 represents the series parasitic resistance of the first switch unit (S_1_) while C_1_ is the unit capacitance of the same switch.

It can be easily proven that [10]:

(3) $$Q_{c}=(C_{Par}+C_{Var1}+CB+15C_{1})\frac{2}{\omega_{0}({C_{Var1}}^{2}R_{Var}+{CB}^{2}R_{C}+15{C_{1}}^{2}R_{on})}$$

The quality factor described in Equation (3) corresponds to the scenario where all capacitors within the capacitor bank are switched ON. When the capacitors are in the OFF state, their quality factor is notably higher than in the ON state. The design involves a trade-off related to the transistor switch width. While increasing the width reduces the switch’s resistance, R_on_, it also adds more parasitic capacitance to the tank circuit, which can narrow the tuning range. A compromise must be achieved between phase noise performance and tuning range which is dictated by the width of the switch transistor. Besides the above challenges and the added design complexity and trade-offs in utilizing a multibit capacitor bank, it is highly beneficial for the VCO’s general performance as the VCO’s gain is largely lowered. As a result, phase noise fluctuations arising from AM-PM conversions are greatly reduced. Furthermore, while increasing the value of C_B_ results in a lower quality factor, as previously discussed, there is a minimum threshold for C_B_ below which the loop gain falls below unity. Hence, a careful balance must be achieved.

Moreover, $R_{S1}$ in Figure 6 is the series parasitic resistance of the primary winding (L_P1_) of transformer T1. L_P2_ is the secondary winding of transformer T1, $R_{S2}$ represents its series parasitic resistance, while C_P_ is the capacitance seen in the input of the frequency doubler.

From Figure 6 the input impedance of transformer T1 can be calculated as below:

(4) $$\;\;Z_{in}=R_{S1}+j\omega L_{P1}+\frac{\omega^{2}k_{1}\sqrt{L_{P1}L_{P2}}}{1+j\omega C_{P}R_{S2}-\omega^{2}L_{P2}C_{P}}$$

It follows that,

(5) $$Q_{L}=\frac{Imag(Z_{in})}{Real(Z_{in})}=A\left\{R_{S1}-\omega^{2}L_{P2}C_{P}{+\omega }^{2}L_{P1}C_{P}R_{S2}\right\}+\omega^{2}k_{1}\sqrt{L_{P1}L_{P2}}-\;\omega^{4}k_{1}\sqrt{L_{P1}L_{P2}}L_{P2}C_{P}-j\omega^{3}k_{1}\sqrt{L_{P1}L_{P2}}C_{P}R_{S2}+jA\{\omega L_{P1}-\omega R_{S1}C_{P}-\;\omega^{2}L_{P2}C_{P}L_{P1}$$

where A = ${\left(1-\omega^{2}L_{P2}C_{P}\right)}^{2}$ + ${\left(C_{P}R_{S2}\omega \right)}^{2}$

Ignoring the high frequency terms of ω ≥ ω^3^ =>

(6) $$Q_{L}≅\frac{A\{\omega L_{P1}-C_{P}R_{S1}R_{S2}\}}{A\left\{R_{S1}-\omega^{2}L_{P2}C_{P}{+\omega }^{2}L_{P1}C_{P}R_{S2}\right\}+\omega^{2}k_{1}\sqrt{L_{P1}L_{P2}}}$$

**Figure 7:**
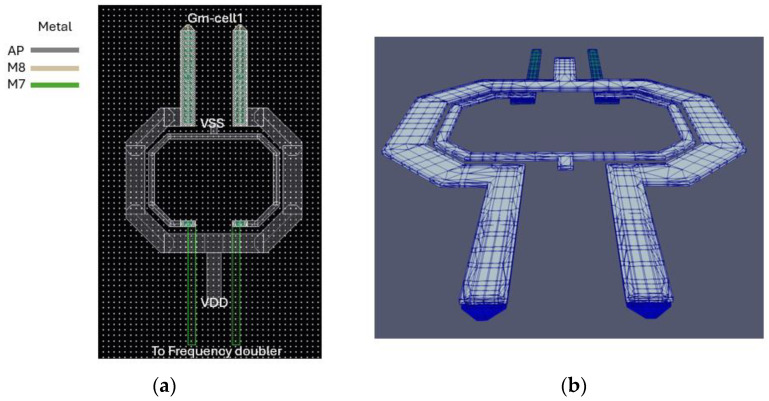
(**a**) 2D view of Transformer T1; (**b**) 3D view of T1 transformer.

**Figure 8:**
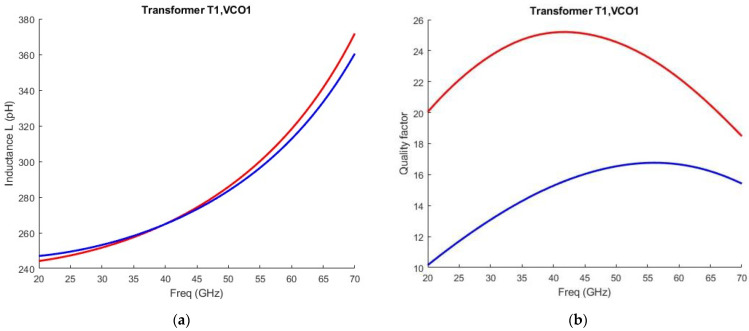
(**a**) Inductance versus frequency of the primary winding (red curve) and secondary winding (blue curve) of Transformer T1; (**b**) Quality factor versus frequency of the primary winding (red curve) and secondary winding (blue curve) of Transformer T1. Both graphs are taken from EXM simulations.

As shown from Equation (6) a larger coupling coefficient leads to a decreased quality factor. In addition to that, both series parasitic resistances must be minimized to improve the quality factor. The transformer T1 is depicted in Figure 7a. It is a dual-tapped structure. The primary winding is a custom one-turn differential coil with a middle tap for biasing purposes, made from Aluminum with a metal width of 16 to reduce the ohmic losses and enlarge the quality factor. The secondary winding is a custom one-turn coil made from Aluminum with a trace width of 5 μm. A larger trace width would increase the quality factor of the secondary coil at the cost of a lower Self-Resonant Frequency (SRF). The use of a differential transformer helps cancel out a portion of the parasitic effects originating from the lossy substrate [31]. The space between the two inductors is set to 4 μm. A larger space between the two inductors would decrease the coupling coefficient(k_1_) leading to an improved quality factor, albeit the signal power delivered to the frequency doubler would be limited. The coupling coefficient reaches 0.47 at 30 GHz. The inductance of both windings, alongside their respective quality factors, is illustrated in Figure 8. The results are obtained from EMX simulations.

**Figure 9:**
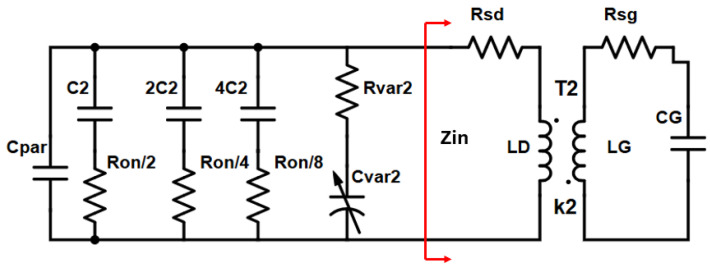
LC-Tank of the high-band oscillator (VCO_2_).

Similarly, Figure 9 illustrates the tank of the high band VCO (Figure 1, VCO_2_) core.

In Figure 9, C_var2_ stands for the varactor capacitance, R_var2_ is the series parasitic resistance of the varactor, C_par_ is the parasitic capacitance of the tank including the parasitic capacitance of the cross-coupled pair (M_3_, M_4_), the series combination of gate capacitance, C_GS_, of the buffer transistors (M_5_, M_6_) and the AC-coupling capacitance (C_CC1_, C_CC2_), the parasitic capacitance of the buffer biasing resistors R_B_ to the substrate and the parasitic capacitance of the switches (B_1_, B_3_). Furthermore, Ron/2 represents the series parasitic resistance of the first switch unit (B_1_) while C_2_ is the unit capacitance of the same switch.

It can be easily proven that:

(7) $$Q_{c}=(C_{Par}+C_{Var2}+7C_{2})\frac{2}{\omega_{0}({C_{Var2}}^{2}R_{Var2}+7{C_{2}}^{2}R_{on})}$$

The quality factor of the capacitive components connected to the tank of the high band VCO (Q_C_) is calculated based on the same principles as in Equation (3).

Moreover, R_sd_ in Figure 9 is the series parasitic resistance of the primary winding (L_D_) of transformer T2. L_G_ is the secondary winding of transformer T2, R_sg_ represents its series parasitic resistance and C_G_ is the capacitance seen at the input of the cross-coupled pair (M_3_, M_4_).

The quality factor of the transformer is given below as in Equation (6)

(8) $$Q_{L}≅\frac{A\{\omega L_{D}-C_{G}R_{sd}R_{sg}\}}{A\left\{R_{sd}-\omega^{2}L_{G}C_{G}{+\omega }^{2}L_{D}C_{G}R_{sg}\right\}+\omega^{2}k_{2}\sqrt{L_{D}L_{G}}}$$

where A = ${\left(1-\omega^{2}L_{G}C_{G}\right)}^{2}$ + ${\left(C_{G}R_{sg}\omega \right)}^{2}$.

**Figure 10:**
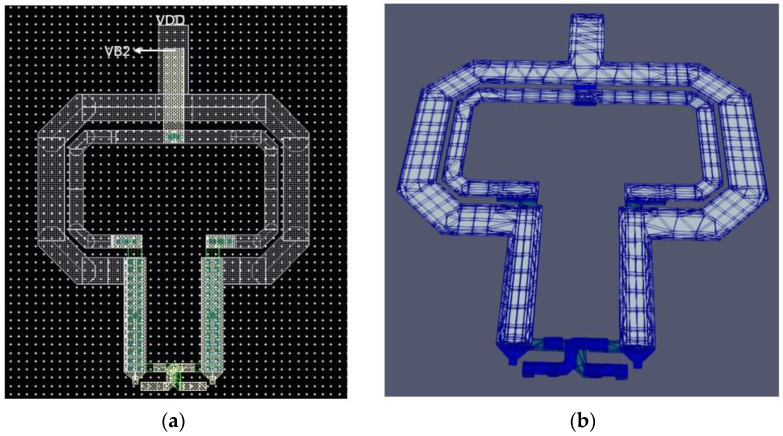
(**a**) Two-dimensional view of Transformer T2; (**b**) 3D view of T2 transformer.

**Figure 11:**
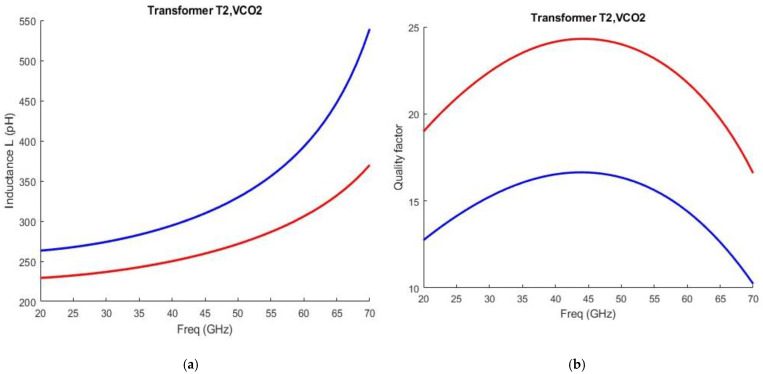
(**a**) Inductance versus frequency of the primary winding (red curve) and secondary winding (blue curve) of Transformer T2; (**b**) Quality factor versus frequency of the primary winding (red curve) and secondary winding (blue curve) of Transformer T2. Both graphs are taken from EXM simulations.

The stacked transformer T2 is shown in Figure 10. It is crucial for the performance of the high-band VCO. The primary winding is connected to the drains of the cross-coupled pair and is a custom one-turn differential structure made of Aluminum at the top and a parallel combination of Aluminum and Metal-8 in the feed lines. It has a trace width of 14 μm. The secondary winding is connected at the gates of the cross-coupled pair. Underneath the feed lines of the primary winding, the secondary winding is made of Metal-7 while in the upper part it is made of Aluminum. It has a trace width of 8 μm. There is a tradeoff between the quality factor and the loop-gain regarding transformer-coupled class-C VCOs. A larger coupling between the two windings would facilitate the start-up condition, although it will decrease the quality factor of the tank. The coupling coefficient reaches 0.55 at 30 GHz. The inductance of both windings, alongside their respective quality factors as obtained from EMX simulations, is illustrated in Figure 11.

The characteristics of the capacitor banks utilized in both VCO cores alongside the parameters of both transformers (T1, T2) at 30 GHz are cited in Table 2. The capacitors are rotative metal-oxide-metal capacitors (RTMOM) from the PDK and are suitable for high frequency design. The parameters of the transformers have been extracted using Cadence’s EMX.

#### 2.2.4. Adaptive Bias and VCO Core Select Circuitry

**Figure 12:**
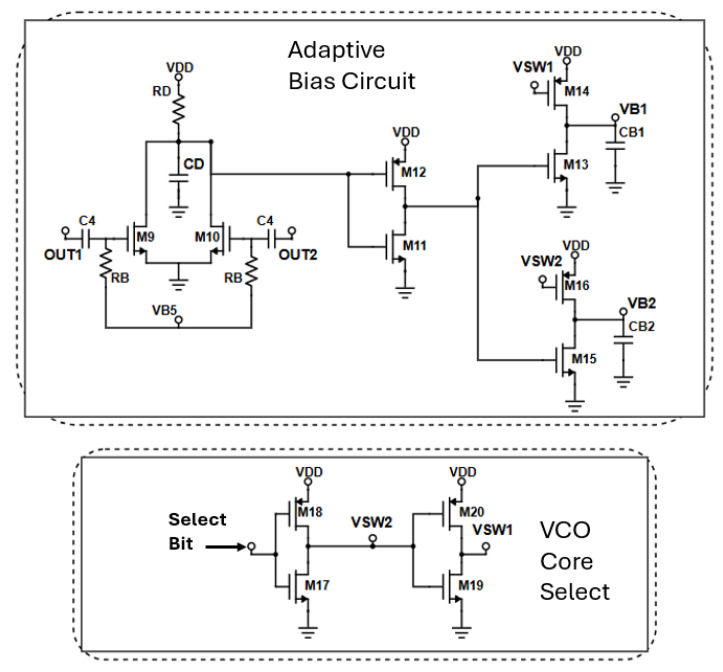
At the top of the figure, the adaptive bias circuitry is illustrated while at the bottom the VCO core select architecture is shown.

**Figure 13:**
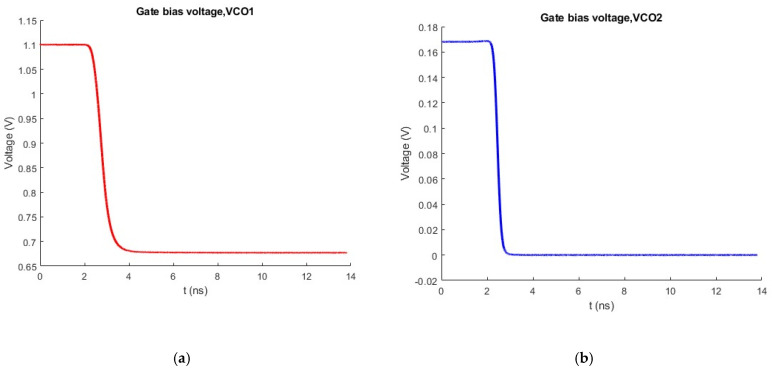
(**a**) Transient signal at node V_B1_; (**b**) Transient signal at node V_B2_.

To alleviate the inherent start-up problem in class-C oscillators [32], a dynamic adaptive bias structure is incorporated in the proposed architecture. In Figure 12 the adaptive bias topology alongside the VCO select circuitry is depicted. The oscillation detector mainly consists of transistors M_9_ and M_10_. The fundamental RF signal is differentially AC coupled to their gates. The transistor pair is biased below V_T_ in the subthreshold region. In the absence of oscillations, the voltage at the common drain terminal of the detector pair is equal to the supply voltage, as no current is flowing through resistor R_D_. Consequently, the output voltage of the CMOS inverter (M_11_, M_12_) is zero, switching M_13_, M_15_ OFF. In the case where VCO_1_ is selected, the ‘Select bit’ is low. As a result, the voltage at node ‘V_SW1_′ is low, driving V_B1_ to the supply voltage (1.1 V) and thus starting the oscillation in strong class-B mode. The large voltage at the gates of the cross-coupled pair creates robust starting conditions. At the same time, the node ‘V_SW2_’ is high, driving V_B2_ to zero. When the low-band VCO core starts oscillating, the voltage at the gates of the detector pair (M_9_, M_10_) starts to increase. Consequently, the voltage at their common drain terminal decreases and the voltage after the inverter increases turning ON NMOS transistors M_13_ and M_15._ By properly selecting the width and the length of M_13_ and M_14_, the appropriate DC voltage for class-C operation can be achieved. In the proposed design, each VCO core starts oscillating with a gate voltage equal to 1.1 V, while for the class-C operation it is lowered to 0.66 V. For the activation of the high-band VCO core the same procedure is followed by setting the ‘Select Bit’ to 1.1 V. Figure 13 demonstrates the bias voltage of both cross-coupled pairs when the low band VCO is activated. The bias circuitry slightly affects the phase noise performance of the low-band VCO core as it is directly tied to its resonance tank. A minimal deterioration results from coupling capacitors(C_4_) due to their parallel parasitic resistance. For this reason, these capacitors have been made as small as possible without compromising the voltage swing at the common drain terminal of the push-push pair. Despite the complexity it adds to the circuit, the proposed biasing topology significantly mitigates the start-up problem, while also helping to maintain the power consumption at low levels.

The values of the proposed adaptive bias/VCO core select circuitry are given in Table 3. The capacitors are rotative metal-oxide-metal capacitors (RTMOM) from the PDK.

### 2.3. Gain-Boosted Frequency Doubler

**Figure 14:**
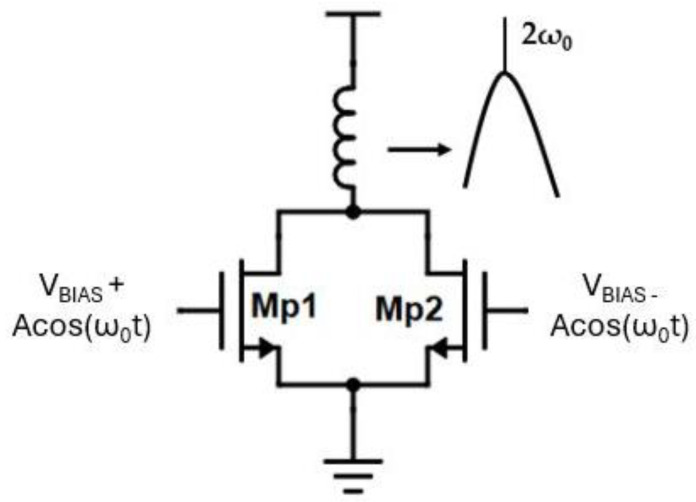
General push-push frequency doubler. The output resonates at the second harmonic to boost the conversion gain.

A 60-GHz VCO can be realized by employing a push-push pair as a frequency doubler. The traditional approach for the design of the push-push frequency doubler is illustrated in Figure 14 [33]. The fundamental signal is applied differentially to the gates of the push-push pair (M_p1_, M_p2_), while the second harmonic signal is collected from the common drain terminal where the anti-phase fundamental tones cancel each other out.

Writing the drain current of a MOS transistor as [34]:

(9) $$i_{d1}\left(t\right)=i_{0}+{gm}_{1}V_{gs}+{gm}_{2}{V_{gs}}^{2}+\cdots$$

For the current at the common drain terminal, it follows that:

(10) $$i_{d}\left(t\right)=i_{d1}\left(t\right)+i_{d2}\left(t\right)i_{0}=2(i_{0}+{gm}_{2}{V_{gs}}^{2}+\cdots )$$

In the case of a fundamental signal of Acos(ωt) at the primary winding of T1, neglecting the bias voltage it follows that: $V_{gs}$ = ak_1_Acos(ωt)

(11) $$i_{d}\left(t\right)\approx 2\left(i_{0}+{gm}_{2}{(ak_{1}A}^{2})\right)+{gm}_{2}{\left(ak_{1}A\right)}^{2}cos(2\omega t)$$

where a *=* $\frac{C_{4}}{C_{4}+C_{GS}}$ and $k_{1}$ is the coupling coefficient of Transformer T1.

**Figure 15:**
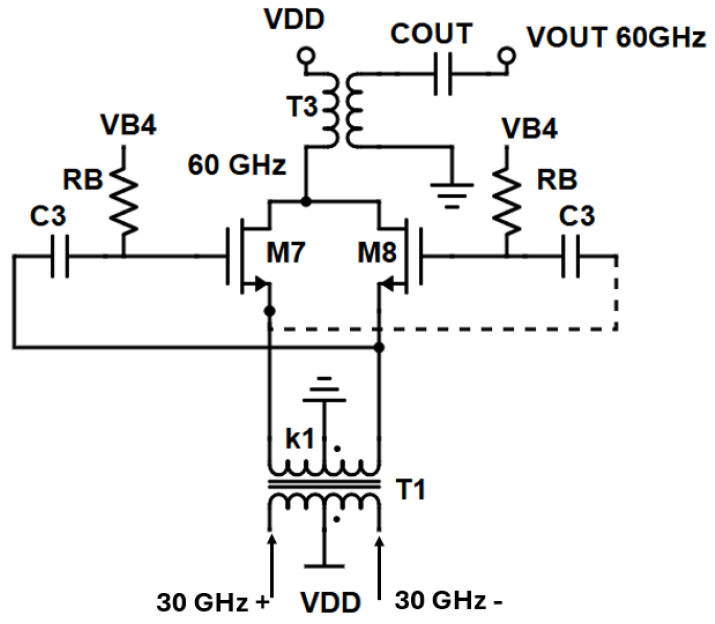
Gain boosted frequency doubler implemented in the design.

The proposed architecture of the frequency doubler is altered from the traditional described in [33]. A gain-boosting topology [35] has been utilized and is illustrated in Figure 15. Instead of directly tying the 30 GHz signal to the gates of the push-push pair (M_7_–M_8_) the signal is AC-coupled to the gates via an RC-network formed by capacitor C_3_ and biasing resistor R_B_, and to the sources of the push-push pair via a cross-coupled type connection. The fundamental RF signal from either the low band or the high band VCO is directly driven through the secondary coil of transformer T1 to the sources of the push-push transistors as well as through capacitive coupling to the gates of the push-push pair. The pair is biased through an RC biasing network (R_B_, C_3_) near the subthreshold to boost the conversion gain of the second harmonic. A transformer (T3) whose primary winding resonates with the parasitic capacitance at the common drain terminal of the push-push pair at the second harmonic alongside a capacitor for matching purposes (C_out_) outputs the signal to the output pads.

By employing the gain boosting technique:

(12) $$V_{gs}=V_{g}-V_{s}=ak1Acos(\omega t)-(-k1Acos(\omega t))=aA(k1+1)cos(\omega t)$$

and thus:

(13) $$\;\;i_{d}\left(t\right)\approx 2\left(i_{0}+{gm}_{2}{\left(aA(k_{1}+1\right)}^{2})\right)+{gm}_{2}{\left(aA\left(k_{1}+1\right)\right)}^{2}cos(2\omega t)$$

indicating that the second harmonic AC signal is boosted by $\frac{k_{1}+1}{k_{1}}$ times.

A larger coupling coefficient (k_1_) boosts the conversion gain as seen in the above Equations (11) and (13) at the cost of a degraded quality factor of the low band VCO tank.

**Figure 16:**
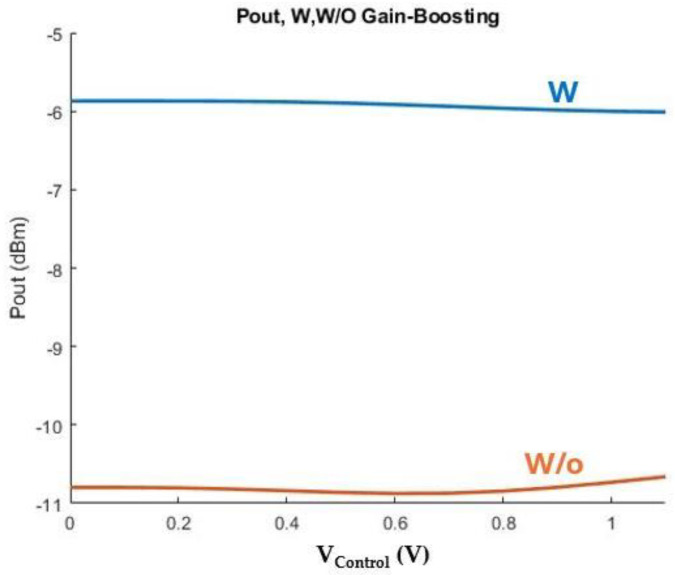
Output power across control voltage (V_Control_) in the absence of the gain-boosting technique (orange line) and with the proposed design (blue line).

As seen in Figure 16, regarding the low band VCO core in the frequency band with the digital code ’0110’ the output power at the output port is increased by more than 4 dBm across the bandwidth of interest, by employing the proposed configuration.

The proposed gain-boosted frequency doubler draws 460 μA from a 1.1 Volt power supply.

In Table 4 the parameters of the frequency doubler are given. The transformer parameters are given at 60 GHz. The coupling coefficient reached 0.66 at the same frequency. The capacitors are rotative metal-oxide-metal capacitors (RTMOM) from the PDK.

**Figure 17:**
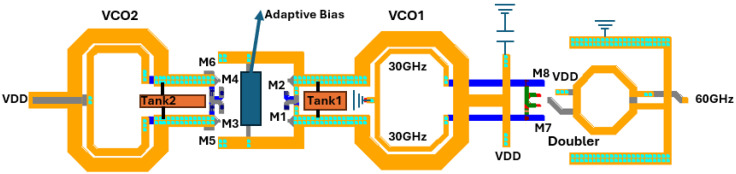
Core Layout of the proposed structure.

A simplified layout of the major blocks is illustrated in Figure 17. The high-band VCO is depicted at the left of Figure 17 and is followed by the buffer. In the middle stands the low band VCO whose tank transformer secondary winding propagates the fundamental signal to the high frequency doubler. The adaptive bias circuitry is located in the feed lines of the primary winding of VCO_1_.

## 3. Post-Layout Simulation Results

**Figure 18:**
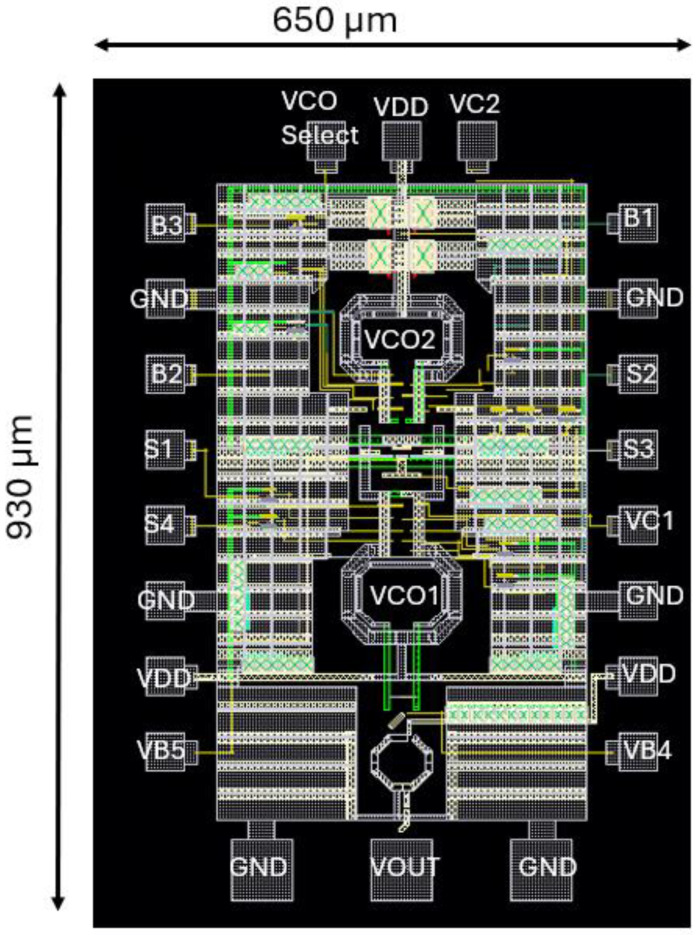
Layout of the proposed structure.

The proposed expansive tuning range 60 GHz VCO structure is designed in a 40 nm TSMC RF CMOS process. In Figure 18 the proposed layout is demonstrated. The total chip area reaches 0.6 (0.65 × 0.93) ${mm}^{2}$ including the two VCO cores, the adaptive bias/VCO select circuitry, the gain-boosted frequency doubler, the DC pads and the RF pads which are located at the top of Figure 18.

At mm-wave frequencies, the impact of parasitics in metal interconnections becomes highly pronounced. Parasitic inductance and capacitance from these interconnections play a crucial role in influencing the oscillation frequency, while parasitic resistance degrades the phase noise performance. One strategy to mitigate parasitic resistance is to use wider metal traces and interconnections, though this comes at the cost of increased parasitic capacitance and a reduced tuning range. A remedy for the resulting unwanted capacitance is to utilize top metals which are located furthest from the lossy substrate. All the critical interconnections have been made by Aluminum (R_Sheet_ = 21.4 mΩ/sq) the top and thickest metal that technology provides. This approach leads to metal paths with less parasitic resistance and less capacitance from the silicon substrate. The critical paths of both VCO cores that contribute to the phase noise performance have been kept as short and as wide as possible to prevent phase noise deterioration. To ensure reliable simulation results, the two-VCO cores alongside the frequency doubler and the crucial metal interconnections have been simulated in Cadence’s EMX as a multiport S parameter structure up to the fourth harmonic.

**Figure 19:**
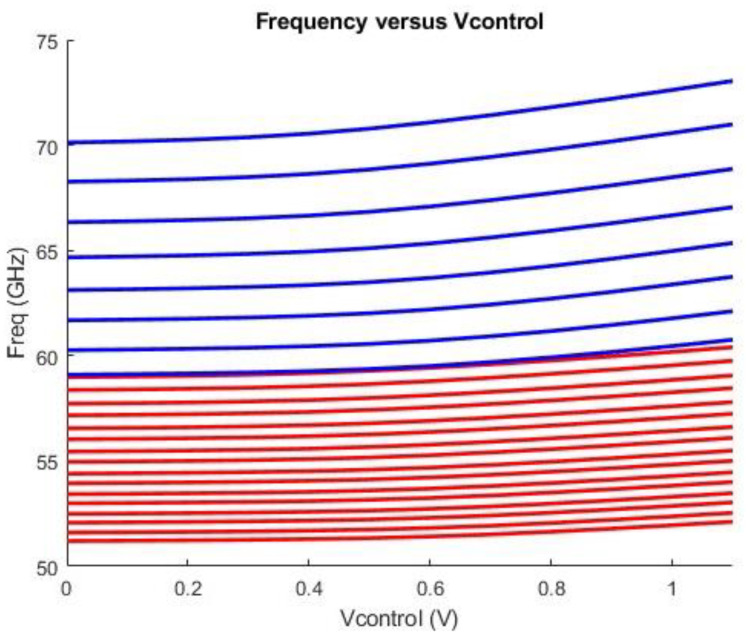
Total VCO bands at the output utilizing second harmonic generation. The red curves correspond to the low band core, while the blue curves refer to the high band core.

Post-layout simulations reveal that the proposed VCO operates across a total frequency range from 50.25 GHz to 73 GHz, resulting in a tuning range of approximately 37%. This range is divided into twenty-four oscillation bands, where VCO_1_ contributes sixteen oscillation bands and VCO_2_ eight oscillation bands. The two groups of bands overlap to avoid dead zones. The varactor’s control voltage in both VCO cores was swept from 0 to 1.1 V to achieve this range. In all subsequent figures, the oscillation bands are grouped in descending order of frequency. Figure 19 showcases the complete set of frequency bands.

**Figure 20:**
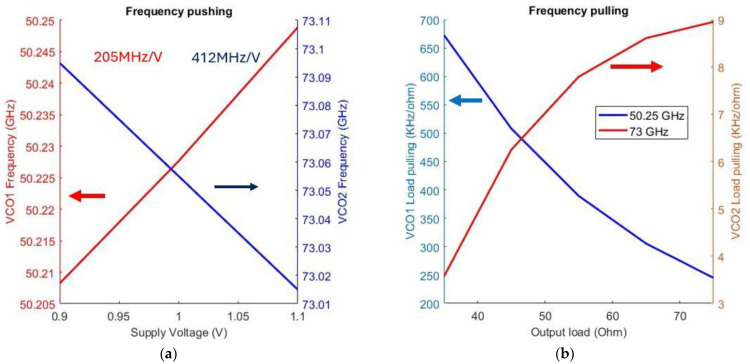
(**a**) Simulated supply pushing where the red line corresponds to the lowest frequency point of VCO_1_ and the blue line corresponds to the highest frequency of oscillation of VCO_2_; (**b**) simulated load pulling in KHz/V where the red line corresponds to the lowest frequency point of (VCO_1_ and the blue line corresponds to the highest frequency of oscillation of VCO_2_.

Fluctuations in the supply voltage influence the parasitic capacitance of the active devices, resulting in variations in the oscillation frequency. To mitigate this effect, the proposed design employs a Class-C topology for both VCO tanks, which minimizes the conduction angle of the cross-coupled switches. Figure 20a illustrates the relationship between the oscillation frequency of the proposed structure and changes in the supply voltage. As illustrated, the high band VCO core is more susceptible to variations from the power supply voltage as it presents a higher VCO gain (K_VCO_) than the low band VCO core. This is expected as it utilizes a larger varactor than the low-band VCO core. The use of a low-dropout regulator (LDO) would stabilize the power supply and further reduce the power supply pushing. In addition, frequency variations arising from changes in the load impedance at the output port are illustrated in Figure 20b. As shown, the suggested design is robust against load variations, as both VCO cores are isolated from the load.

**Figure 21:**
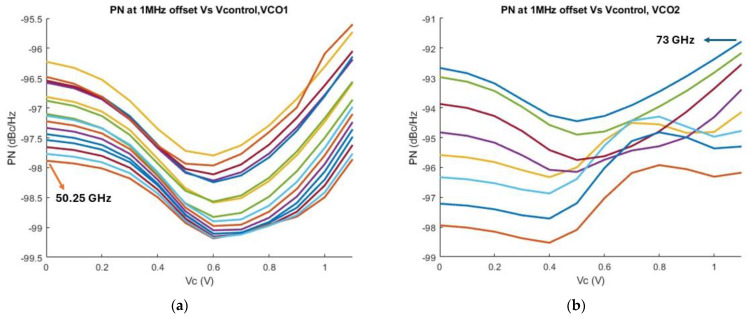
(**a**) Simulated Phase Noise levels of the low band VCO at 1 MHz frequency offset away from carrier versus control voltage; (**b**) Simulated Phase Noise of the high band VCO at 1 MHz frequency offset versus control voltage.

The phase noise of the output at 1 MHz frequency offset is illustrated in Figure 21. The output of the proposed topology is connected to a 50-ohm port. In Figure 21a the phase noise performance at 1 MHz offset of the low band VCO core is shown, while Figure 21b depicts the phase noise levels of the high band VCO. The phase noise of the low band VCO varies from −99.2 dBc/Hz to −95.55 dBc/Hz at 1 MHz offset from the carrier frequency, while the phase noise levels of the high band VCO show a nearly 6 dB variation from −98.2 dBc/Hz to −92.1 dBc/Hz at 1 MHz offset.

**Figure 22:**
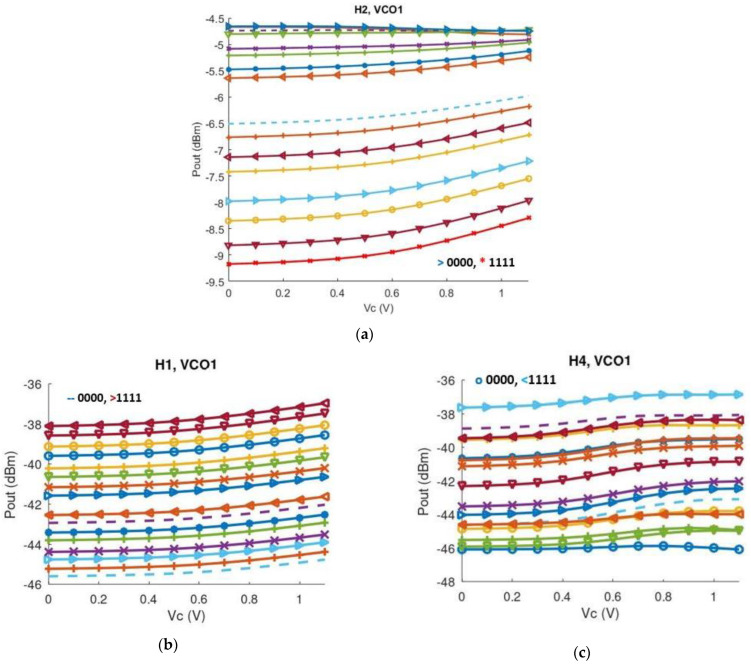
(**a**) Second harmonic (H2) output power levels of the low band VCO core across all sixteen bands versus V_CONTROL_. The code ‘0000’ corresponds to the highest oscillation frequency; (**b**) First harmonic (H1) output power levels of the low band VCO core across all sixteen bands versus V_CONTROL_; (**c**) Fourth harmonic (H4) output power levels of the low band VCO core across all sixteen bands versus V_CONTROL_.

**Figure 23:**
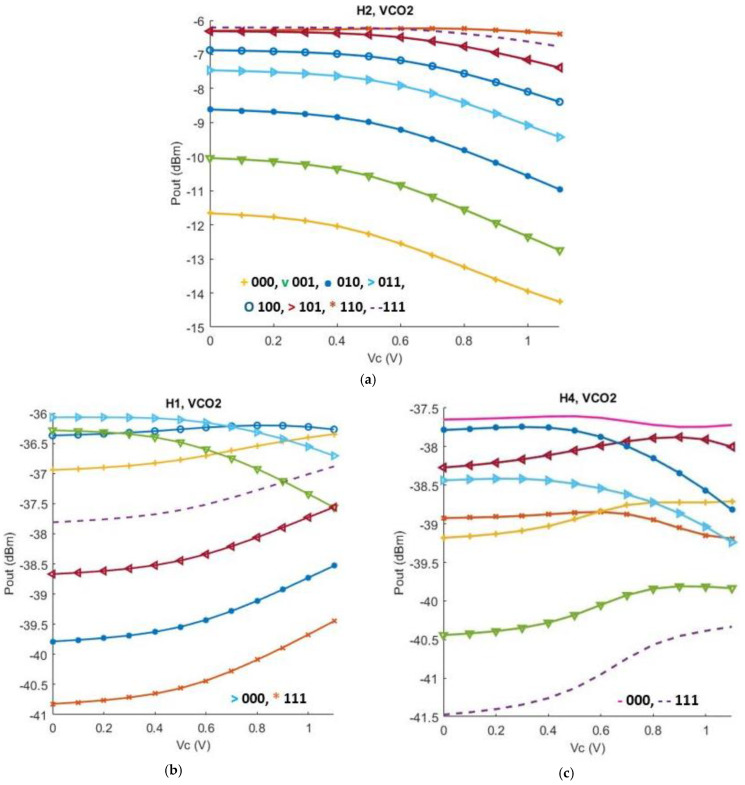
(**a**) Second harmonic (H2) output power levels of the high band VCO core across all eight bands versus V_Control_. The code ‘000’ corresponds to the highest oscillation frequency; (**b**) First harmonic (H1) output power levels of the high band VCO core across all eight bands versus V_CONTROL_; (**c**) Fourth harmonic (H4) output power levels of the high band VCO core across all eight bands versus V_CONTROL_.

Figure 22a shows the output power of the low band VCO core including the desired second harmonic alongside the first, third and fourth harmonic, delivered to a 50-ohm port across all sixteen frequency bands. In addition to that, the first and the fourth undesired harmonics are illustrated in Figure 22b,c. In Figure 23 the same parameters are illustrated regarding the high band VCO core. Across the whole bandwidth, the proposed structure delivers between −4.8 and −14.2 dBm, while undesired harmonics are greatly suppressed.

Table 5 provides a summary of the phase noise levels at a 1 MHz frequency offset from the carrier, alongside the delivered output power across various corners and temperature variations. The Fast-Fast (FF@27 °C) and Slow-Slow (SS@27 °C) corners are compared with the typical case (TT@27 °C). In the slow-slow process, the frequency varies from 47.5 to 71.2 GHz, accompanied by phase noise levels ranging from −96.5 -to −91.3 dBc/Hz at 1 MHz offset from 47.5 and 71 GHz respectively. Output power reaches −15.3 dBm and −17.2 dBm at the two frequency ends. In the fast-fast process, the frequency spans from 53.7 to 75.3 GHz and phase noise levels vary between −97.8 and −93 dBc/Hz at 1 MHz offset from the two frequency ends respectively. Furthermore, the output power is higher and reaches −8.2 dBm at the lower frequency limit and −13.5 dBm at the upper frequency limit. Finally, in higher temperatures the oscillation slightly frequency decreases, phase noise deteriorates, and output power is reduced. The opposite trend is observed at lower temperatures.

The total power dissipation of the chip reaches 7.3 mW when VCO_1_ is activated and 11.3 mW when VCO_2_ is activated. The difference in the power dissipation results from the buffer stage used in the operation of VCO_2_.

## 4. Discussion

Table 6 displays a comparison of the proposed VCO structure with state-of-the-art designs operating within the same frequency range. The proposed VCO achieves a Figure of Merit (FOM) of −181.7 dBc/Hz and a Figure of Merit with Tuning (FOM_T_) of −192.6 dBc/Hz, both measured at a 1 MHz offset from the oscillation frequency of 61.6 GHz, which represents the central operating frequency. The proposed structure demonstrates a substantial tuning range and higher output power levels when compared to similar designs, exhibiting competitive phase noise levels while preserving energy consumption. Due to its performance, the proposed architecture is well suited for integration into transceivers for applications concerning short-range sensing and wireless communication. It provides an extensive tuning range and moderate phase noise levels while delivering sufficient output power. The output power levels are adequate to avoid the need for multiple additional power-consuming buffers, whether for driving the mixer and frequency divider in communication systems or the power amplifier, the divider and the down-conversion mixer in radar sensors. A future line of research would be to fabricate the proposed chip to verify its performance. Addressing the performance of the proposed design in real-world fabrication would provide deeper insights. Factors such as process variations, inconsistencies in metal thickness and unwanted coupling between layers can significantly impact circuit performance. Future work could also explore optimizing the resonance tank of the frequency doubling circuit by inducing multiple resonances across the whole bandwidth to improve further the conversion gain and provide higher output power. Additionally, improving the tuning linearity of both VCO cores would prove beneficial for the phase noise performance.

## 5. Conclusions

This article introduced an extensive tuning range dual-core class-C 60 GHz VCO in 40 nm bulk CMOS process. It presented a clear design strategy for the implementation of the proposed structure. The design methodology can be easily applied to more advanced process nodes such as 28-nm and 22-nm CMOS processes. The proposed architecture features a switchless dual core class-C structure, an adaptive bias circuitry and a gain-boosting frequency doubler. The output frequency ranges from 50.25 GHz to 73 GHz. The combination of the two VCO cores results in a substantial tuning span, competitive phase noise levels and less than 11.3 mW of power dissipation. The presence of the adaptive bias circuitry ensures proper start-up and transition to class-C operation. Owing to the gain-boosted frequency doubler, the output power is moderate despite the large tuning range. Due to its state-of-the-art performance, it can be considered a highly promising candidate for integration in 60 GHz transceivers.

## Figures and Tables

**Table 1:** Fundamental VCO parameters.

Parameter	Value
W_M1-M4_	18 × 1 μm
W_M5-M6_	22 × 1 μm
L	40 nm
C_B_	82 fF
R_B_	4 K
V_B3_	570 mV
C_CC1_	42 fF

**Table 2:** Parameters of LC tanks.

LC Tanks Parameters	Values
C_1_, C_2_	15.3 fF, 24.7 fF
W_Switch,S1_, W_Switch,B1_	12 × 1 μm, 24 × 1 μm
L_Switch,S1–S4_, L_Switch,B1–B3_	40 nm
C_VAR1_, C_VAR2_@V_GS_ = 0	30 fF, 36 fF
T1: L_P1_, L_P2_, Q_P1_, Q_P2_	245 pH, 247 pH, 24, 13.7
T2: L_D_, L_G_, Q_D_, Q_G_	240 pH, 265 pH, 22.1, 13.4

**Table 3:** The parameters of the topology in Figure 12.

Parameter	Value	Parameter	Value
C_4_	42.5 fF	W_M12,_ W_M18, M20_	32 × 5 μm, 10 × 3 μm
W_M9,M10,WM13,M15_	12 × 1 μm, 22 × 2 μm	W_M14, M16_	30 × 5 μm
W_M11,M17,M19_	10 × 1 μm	L_M13-M16_	90 nm
R_D_, R_B_	4.6 K, 6 K	C_B1-B2_, C_D_	4 pF, 3 pF

**Table 4:** Frequency doubler parameters.

Parameter	Value
W_M7, M8_	8 × 1 μm
L_M7, M8_	40 nm
V_B4_	0.4 V
C_3_	75 fF
RB	3 K
C_out_	35 fF
T3:L_P_/Q_P_ at 60 GHz	210 pH/17
T3:L_P_/Q_P_ at 60 GHz	196 pH/14

**Table 5:** Corner Simulations results.

Corners	Frequency (GHz)	PN 1 MHz	PN 1 MHz	Pout dBm.	Pout dBm.
TT	50.25–73	−97.9	−92.1	−9.2	−14.2
SS	47.5–71.2	−96.5	−91.3	−15.3	−17.2
FF	53.7–75.3	−97.8	−93	−8.2	−13.5
TT @ 120 °C	49.5–72.8	−94.8	−90.2	−12	−16.9
TT @ −40 °C	50.9–73.3	−98	−92.8	−7.9	−12.9

**Table 6:** Comparison with State-of-the-art 60 GHz CMOS VCOs.

Reference	Hardware	F_REQ_ (GHz)	PN [dBc/Hz] @ 1 MHz	P_DC,VCO_ (mW)	FTR (%)	Pout (dBm)	$\mathrm{Area}\;({mm}^{2}$)	FOM *	${FOM}_{T}$*	Process
[17]	measured	60.85	−99.8	11.7	15.6	-	0.12 ^1^	−184.1	−188	28 nm CMOS
[21]	measured	61.3	−94.9	8.4	9	−10	0.009 ^1^	−183.4	−180.5	65 nm CMOS
[22]	measured	59	−95	15.3	13.4	−4	0.12 ^1^	−178	−180.5	28 nm CMOS
[23]	Post- layout	60	−93.5	4.2	18.2	−12	0.07 ^1^	−183	−188	65 nm CMOS
[24]	measured	61.4	−96.9	20	18	−22.6	0.08 ^1^	−180	−186	45 nm SOI
[28]	measured	58.9	−92.5	4.7	18.5	-	0.17 ^1^	−181.7	−187.1	65 nm CMOS
[36]	Post- layout	62.4	−97.2	16	10.58	-	0.052 ^1^	−181.2	−181.5	40 nm CMOS
[37]	measured	60	−87	11	16	−23	0.02	−173	−177	28 nm CMOS
**This work**	**Post-** **layout**	**61.6**	**−96.7**	**11.3 ^2^**	**36.92**	**−8.4**	**0.1 ^1^**	**−181.7**	**−192.6**	**40 nm CMOS**

^1^ core area, ^2^ maximum VCO power consumption, * FOM = $L\left(\Delta_{f}\right)$ − 20log$\left(\frac{f_{0}}{\Delta_{f}}\right)$ + 10log$\left(\frac{P_{dc}}{1mW}\right)$, * ${FOM}_{T}$ = $L\left(\Delta_{f}\right)$ − 20log$\left(\frac{f_{0}}{\Delta_{f}}\frac{TR[\%]}{10}\right)$ + 10log$\left(\frac{P_{dc}}{1mW}\right)$.

## Data Availability

Data are contained within the article.
